# Supplementary material for: Comparative Density Functional Theory Study of Magnetic Exchange Couplings in Dinuclear Transition-Metal Complexes
Source: J Chem Theory Comput. 2023 Aug 15;19(17):5760–72. doi: 10.1021/acs.jctc.3c00336 (PMC10500985; doi:10.1021/acs.jctc.3c00336)
Supplement: Supplementary file 1 — ct3c00336_si_001.pdf [file ct3c00336_si_001.pdf]

Supporting Information for Comparative Density Functional Theory Study of Magnetic Exchange Coupling in Di-nuclear Transition Metal Complexes

Henry C. Fitzhugh[1*], James W. Furness[1], Mark R. Pederson[2], Juan E. Peralta[3], and Jianwei Sun[1*]
[1]*Department of Physics and Engineering Physics, Tulane University, New Orleans, Louisiana 70118, USA*
[2]*Department of Physics, the University of Texas at El Paso, El Paso, Texas 79968, USA*
[3]*Department of Physics and Science of Advanced Materials,*
Central Michigan University, Mount Pleasant, Michigan 48859, USA.
(Dated: August 11, 2023)

* hfitzhug@tulane.edu, jsun@tulane.edu

CONTENTS

I. Natural Population Analysis S3

II. Density Difference Plots S5

III. Spin Density and Spin Electron Localization Function S6

IV. Additional Computational Settings S8

V. Magnetic Hamiltonians and Data as given in experimental references S9

I. NATURAL POPULATION ANALYSIS

Table S1. Natural Population Analysis (NPA) from the four Mn_2 complexes where the coordination and oxidation is the same for each Mn atom. Both the initial high spin states and the converged 'spin flipped' states were analyzed. Some quantities from manganese atoms are only provided as an average, Mn_a. *apsd* - atomic population from spin density, *NC* - natural charge, *3d* - population in 3d natural orbital, *val* - population in valence orbitals, *TC* - total charge, $J_{err} = J_{exp.} - J_{func.}$ Further information regarding quantities from NPA can be found in the Turbomole 7.6 User Manual and the references therein.

		HS								BS								
		Mn1	Mn2	Mn_a				Oxy		Mn1	Mn2	Mn_a				Oxy		
		apsd	apsd	NC	3d	val	TC	apsd	NC	apsd	apsd	NC	3d	val	TC	apsd	NC	J_{err}
1-TIPFAZ	PBE	2.81	2.82	1.38	5.20	5.41	23.62	0.15	-0.81	−2.62	2.71	1.36	5.23	5.44	23.64	0.00	-0.78	−28.6
	SCAN	2.83	2.84	1.52	5.00	5.21	23.48	0.15	-0.89	−2.68	2.76	1.52	5.01	5.22	23.48	0.00	-0.88	−14.0
	r^2SCAN	2.94	2.95	1.51	5.01	5.22	23.49	0.09	-0.89	−2.77	2.86	1.50	5.03	5.24	23.50	−0.01	-0.88	10.0
	TPSSh	2.91	2.92	1.50	5.02	5.23	23.49	0.11	-0.88	−2.74	2.84	1.50	5.03	5.25	23.50	−0.01	-0.87	4.5
	PBE0	3.06	3.08	1.60	4.86	5.08	23.40	0.04	-0.93	−2.92	3.00	1.60	4.88	5.10	23.40	−0.03	-0.93	31.5
	lh07t-SVWN	3.05	3.07	1.55	4.94	5.16	23.45	0.05	-0.91	−2.91	2.97	1.54	4.96	5.17	23.46	−0.02	-0.91	34.9
	lh07s-SVWN	3.03	3.05	1.53	4.97	5.19	23.46	0.05	-0.90	−2.90	2.96	1.52	4.99	5.20	23.47	−0.02	-0.89	32.4
	lh12ct-ssir	3.02	3.03	1.58	4.90	5.12	23.42	0.06	-0.92	−2.89	2.95	1.58	4.92	5.13	23.42	−0.03	-0.92	31.3
	lh12ct-ssif	3.03	3.05	1.60	4.88	5.09	23.40	0.05	-0.93	−2.92	2.98	1.59	4.89	5.11	23.41	−0.03	-0.93	34.9
	lh14t-calPBE	3.03	3.04	1.56	4.93	5.14	23.43	0.00	-0.91	−2.88	2.95	1.55	4.94	5.16	23.44	−0.02	-0.91	28.1
	lh20t	2.99	3.00	1.60	4.87	5.08	23.39	0.03	-0.93	−2.86	2.90	1.60	4.88	5.09	23.40	−0.04	-0.93	25.9
2-GEFKAD	PBE	3.71	3.72	1.37	5.29	5.55	23.63	0.14	-0.80	−3.48	3.53	1.34	5.34	5.59	23.68	−0.03	-0.76	−55.9
	SCAN	3.78	3.79	1.49	5.17	5.41	23.51	0.10	-0.88	−3.61	3.66	1.47	5.19	5.44	23.53	−0.04	-0.87	−16.2
	r^2SCAN	3.81	3.82	1.48	5.18	5.42	23.52	0.07	-0.88	−3.64	3.70	1.46	5.21	5.45	23.54	−0.04	-0.87	−3.2
	TPSSh	3.79	3.80	1.48	5.17	5.42	23.52	0.10	-0.88	−3.62	3.67	1.46	5.20	5.45	23.53	−0.04	-0.87	−15.4
	PBE0	3.86	3.87	1.56	5.08	5.33	23.44	0.06	-0.93	−3.71	3.79	1.55	5.10	5.34	23.45	−0.04	-0.93	5.9
	lh07t-SVWN	3.87	3.88	1.52	5.12	5.37	23.48	0.06	-0.91	−3.72	3.79	1.51	5.15	5.39	23.49	−0.05	-0.90	9.6
	lh07s-SVWN	3.87	3.88	1.51	5.14	5.39	23.49	0.06	-0.89	−3.77	3.78	1.49	5.17	5.41	23.51	−0.05	-0.89	6.8
	lh12ct-ssir	3.85	3.86	1.55	5.10	5.34	23.45	0.07	-0.92	−3.70	3.77	1.54	5.12	5.36	23.46	−0.05	-0.92	1.9
	lh12ct-ssif	3.86	3.87	1.56	5.08	5.32	23.44	0.06	-0.93	−3.72	3.78	1.55	5.10	5.34	23.45	−0.05	-0.93	4.6
	lh14t-calPBE	3.85	3.86	1.53	5.12	5.36	23.47	0.06	-0.91	−3.70	3.77	1.52	5.14	5.38	23.48	−0.05	-0.91	5.5
	lh20t	3.81	3.82	1.57	5.08	5.32	23.43	0.08	-0.94	−3.69	3.73	1.55	5.09	5.33	23.44	−0.04	-0.94	−1.7
4-ZEQGOR	PBE	2.74	2.74	1.01	5.52	5.80	23.98	0.20	-0.69	−2.49	2.57	0.97	5.59	5.87	24.03	0.02	-0.63	−105.2
	SCAN	2.74	2.74	1.16	5.33	5.61	23.84	0.22	-0.77	−2.60	2.69	1.13	5.37	5.65	23.87	0.02	-0.73	−1.3
	r^2SCAN	2.86	2.86	1.15	5.35	5.63	23.85	0.16	-0.76	−2.67	2.78	1.12	5.40	5.67	23.88	0.01	-0.72	4.6
	TPSSh	2.83	2.83	1.14	5.35	5.64	23.86	0.17	-0.76	−2.66	2.76	1.11	5.39	5.68	23.89	0.01	-0.72	4.7
	PBE0	3.00	3.00	1.23	5.21	5.50	23.77	0.08	-0.81	−2.81	2.95	1.21	5.24	5.53	23.79	−0.01	-0.78	55.7
	lh07t-SVWN	3.00	3.00	1.18	5.29	5.58	23.82	0.09	-0.79	−2.79	2.92	1.15	5.33	5.62	23.80	0.00	-0.75	29.5
	lh07s-SVWN	2.99	2.99	1.16	5.31	5.60	23.84	0.09	-0.77	−2.77	2.90	1.13	5.36	5.65	23.87	0.00	-0.74	20.7
	lh12ct-ssir	2.98	2.98	1.21	5.25	5.54	23.79	0.09	-0.80	−2.78	2.91	1.18	5.29	5.58	23.82	0.00	-0.77	37.9
	lh12ct-ssif	2.99	2.99	1.22	5.23	5.52	23.78	0.08	-0.81	−2.80	2.93	1.20	5.27	5.56	23.80	−0.01	-0.78	45.2
	lh14t-calPBE	2.98	2.98	1.19	5.27	5.56	23.81	0.10	-0.79	−2.78	2.90	1.17	5.32	5.60	23.83	0.00	-0.76	35.2
	lh20t	2.94	2.94	1.23	5.22	5.50	23.77	0.11	-0.81	−2.77	2.89	1.21	5.25	5.54	23.78	0.00	-0.79	43.6
5-VADDAF	PBE	2.80	2.79	1.20	5.37	5.61	23.80	0.16	-0.72	2.44	−2.37	1.10	5.52	5.76	23.90	−0.02	-0.65	−205.9
	SCAN	2.78	2.79	1.34	5.19	5.43	23.67	0.17	-0.79	2.64	−2.58	1.29	5.28	5.51	23.71	−0.03	-0.76	9.3
	r^2SCAN	2.91	2.90	1.33	5.20	5.44	23.67	0.11	-0.79	2.69	−2.63	1.27	5.30	5.53	23.73	−0.02	-0.75	−9.3
	TPSSh	2.87	2.87	1.32	5.21	5.45	23.68	0.12	-0.78	2.72	−2.63	1.26	5.30	5.54	23.73	−0.04	-0.74	−20.8
	PBE0	3.04	3.03	1.42	5.06	5.31	23.58	0.03	-0.83	2.90	−2.80	1.38	5.13	5.38	23.62	−0.03	-0.80	64.0
	lh07t-SVWN	3.05	3.03	1.36	5.14	5.39	23.60	0.03	-0.81	−2.76	2.88	1.31	5.23	5.47	23.69	−0.04	-0.77	14.7
	lh07s-SVWN	3.03	3.02	1.34	5.17	5.41	23.66	0.04	-0.80	−2.74	2.86	1.29	5.26	5.50	23.71	−0.04	-0.76	13.8
	lh12ct-ssir	3.02	3.01	1.39	5.10	5.35	23.61	0.04	-0.82	−2.76	2.88	1.34	5.18	5.43	23.65	−0.04	-0.79	38.0
	lh12ct-ssif	3.04	3.03	1.41	5.08	5.33	23.59	0.03	-0.83	−2.79	2.90	1.36	5.16	5.40	23.64	−0.04	-0.80	49.2
	lh14t-calPBE	3.02	3.01	1.37	5.13	5.37	23.63	0.04	-0.82	−2.75	2.87	1.32	5.21	5.45	23.67	−0.04	-0.78	19.6
	lh20t	2.98	2.98	1.41	5.07	5.32	23.59	0.05	-0.84	−2.75	2.85	1.37	5.14	5.39	23.63	−0.04	-0.80	44.3

Table S2. Natural Population Analysis for 3-KUVPEW, the mixed-valence complex with Mn1(III) and Mn2(IV). Abbreviations are the same as given in the caption for Table I.

		Mn1					Mn2					Oxy1	mu-di		Oxy2	mu-ac	
		apsd	N.C.	3d	Val	Total	apsd	N.C.	3d	Val	Total	S.D.	N.C.	Total	S.D.	N.C.	Total
HS	PBE	2.64	1.16	5.44	5.67	23.84	3.74	1.41	5.24	5.51	23.59	0.22	−0.76	8.76	0.08	−0.58	8.58
	SCAN	2.61	1.29	5.26	5.49	23.71	3.80	1.54	5.12	5.37	23.46	0.23	−0.83	8.83	0.08	−0.63	8.63
	r^2SCAN	2.76	1.29	5.27	5.49	23.71	3.84	1.53	5.13	5.38	23.47	0.16	−0.83	8.83	0.07	−0.62	8.62
	TPSSh	2.73	1.27	5.28	5.51	23.73	3.81	1.53	5.12	5.38	23.47	0.18	−0.82	8.82	0.07	−0.61	8.61
	PBE0	2.92	1.38	5.12	5.36	23.62	3.90	1.62	5.02	5.28	23.38	0.08	−0.88	8.88	0.06	−0.63	8.63
	lh07t-SVWN	2.91	1.32	5.20	5.43	23.68	3.92	1.58	5.06	5.32	23.42	0.09	−0.85	8.85	0.09	−0.63	8.63
	lh07s-SVWN	2.81	1.50	5.22	5.47	23.50	3.88	1.71	4.98	5.27	23.29	0.14	−0.87	8.87	0.07	−0.69	8.69
	lh12ct-ssirPW92	2.89	1.35	5.16	5.39	23.65	3.88	1.61	5.04	5.29	23.39	0.10	−0.87	8.87	0.06	−0.64	8.64
	lh12ct-ssifPW92	2.91	1.37	5.13	5.37	23.63	3.90	1.63	5.02	5.27	23.37	0.09	−0.88	8.88	0.06	−0.65	8.65
	lh14t-calPBE	2.89	1.33	5.18	5.42	23.67	3.89	1.59	5.06	5.31	23.41	0.10	−0.86	8.86	0.07	−0.63	8.63
	lh20t	2.85	1.37	5.13	5.36	23.63	3.84	1.63	5.02	5.27	23.37	0.12	−0.87	8.88	0.06	−0.65	8.65
BS	PBE	−2.32	1.09	5.53	5.76	23.91	3.60	1.41	5.26	5.52	23.59	−0.19	−0.72	8.72	0.02	−0.59	8.59
	SCAN	−2.40	1.25	5.32	5.55	23.75	3.74	1.54	5.13	5.37	23.46	−0.21	−0.80	8.80	0.02	−0.63	8.63
	r^2SCAN	−2.50	1.24	5.34	5.57	23.46	3.78	1.53	5.14	5.38	23.47	−0.18	−0.80	8.8	0.02	−0.62	8.62
	TPSSh	−2.48	1.23	5.35	5.58	23.77	3.77	1.53	5.13	5.38	23.47	−0.18	−0.79	8.79	0.02	−0.62	8.62
	PBE0	−2.70	1.34	5.18	5.41	23.66	3.89	1.62	5.03	5.28	23.38	−0.14	−0.86	8.86	0.02	−0.64	8.64
	lh07t-SVWN	−2.63	1.27	5.28	5.51	23.73	3.88	1.58	5.08	5.32	23.42	−0.16	−0.83	8.83	0.02	−0.64	8.64
	lh07s-SVWN	−2.62	1.46	5.25	5.51	23.54	3.86	1.70	4.99	5.27	23.30	−0.18	−0.85	8.85	0.02	−0.69	8.69
	lh12ct-ssirPW92	−2.64	1.31	5.23	5.46	23.69	3.86	1.61	5.05	5.29	23.39	−0.15	−0.84	8.84	0.02	−0.65	8.65
	lh12ct-ssifPW92	−2.67	1.33	5.20	5.43	23.67	3.88	1.62	5.03	5.27	23.37	−0.14	−0.85	8.85	0.02	−0.65	8.65
	lh14t-calPBE	−2.62	1.29	5.26	5.49	23.71	3.86	1.59	5.07	5.32	23.41	−0.16	−0.83	8.83	0.02	−0.64	8.64
	lh20t	−2.63	1.34	5.19	5.42	23.66	3.84	1.63	5.03	5.27	23.37	−0.14	−0.86	8.86	0.03	−0.65	8.63

II. DENSITY DIFFERENCE PLOTS

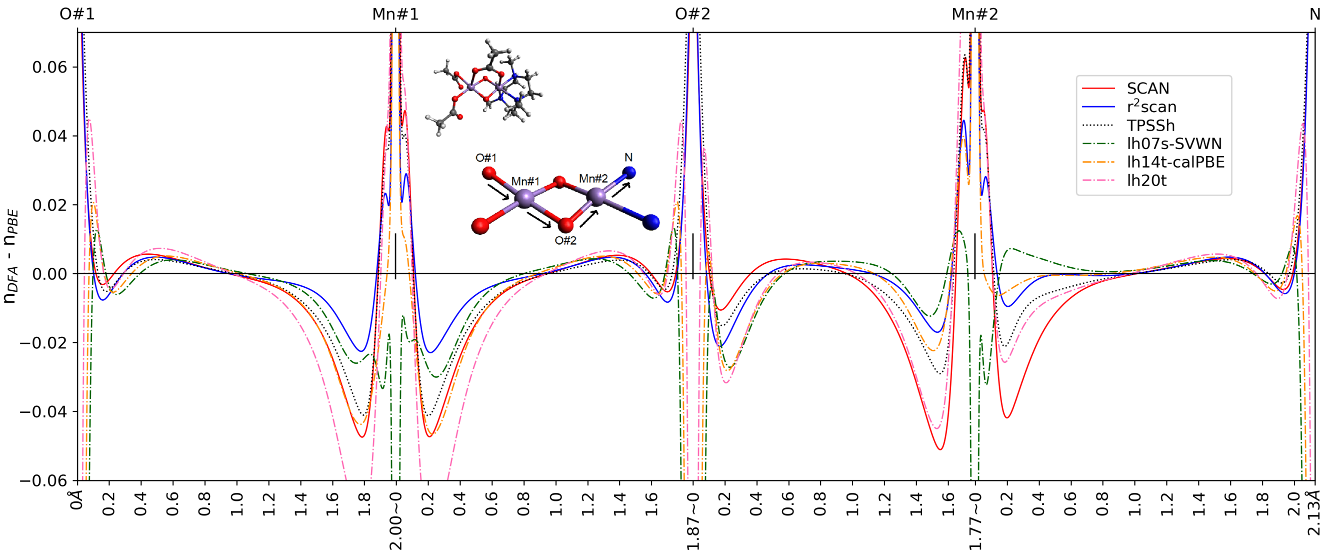

Figure S1. For 3-KUVPEW, the mixed valence dimer, the density from PBE was subtracted from the densities from the other functionals of the study and plotted for a path of line-segments connecting several nuclei within the complex, units are Å. The path through the molecule is shown on the inset map of nuclear positions. From left to right, the distance values reset to zero at each atom along the path.

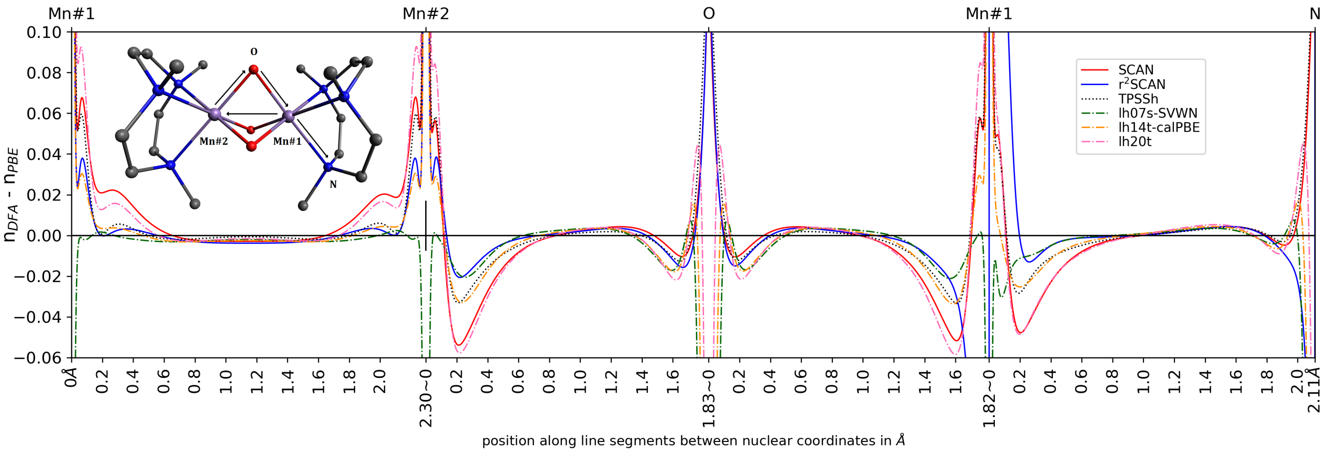

Figure S2. For 5-VADDAF, the density from PBE was subtracted from the densities from the other functionals of the study and plotted for a path of line-segments connecting several nuclei within the complex, units are Å. The path through the molecule is shown on the inset map of nuclear positions. From left to right, the distance values reset to zero at each atom along the path.

III. SPIN DENSITY AND SPIN ELECTRON LOCALIZATION FUNCTION

Spin-density, spin-resolved electron localization function (sELF), and density difference plots demonstrate that unpaired manganese valence electrons are the primary contributors to the *re*localized density in the density difference plots (see FIG. 6 of the primary article). The sELF is non-zero in the valence-orbital region of manganese.

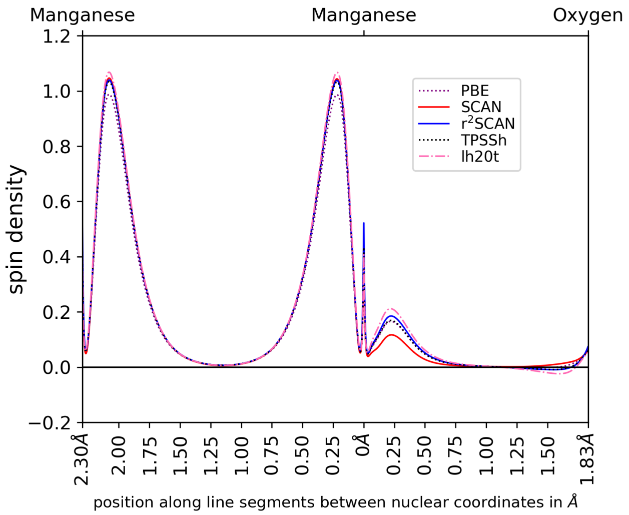

Figure S3. For 5-VADDAF, the linear spin density, sd $= n_\alpha - n_\beta$, of five functionals is shown for the inter-manganese and manganese-oxygen line segments.

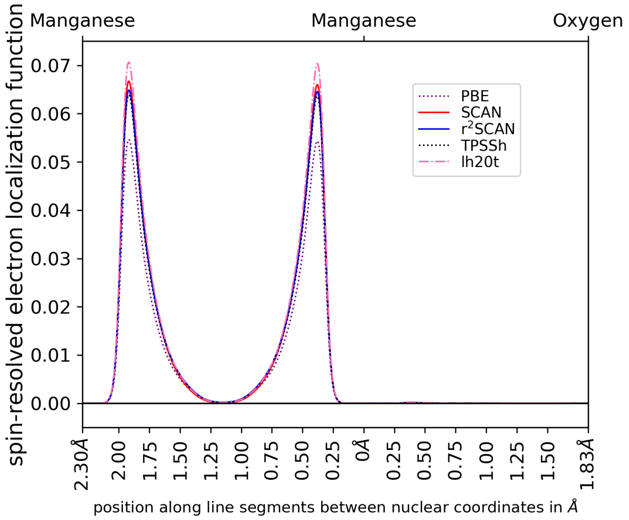

Figure S4. For 5-VADDAF, the spin-resolved electron localization function of five functionals is shown for the inter-manganese and manganese-oxygen line segments. Along the inter-manganese line segment, all functionals produce a peaks approximately 0.37Å from the manganese nuclei. All functionals show a slight non-zero bump from approximately 0.30Å to 0.90Å along the manganese-oxygen line segment.

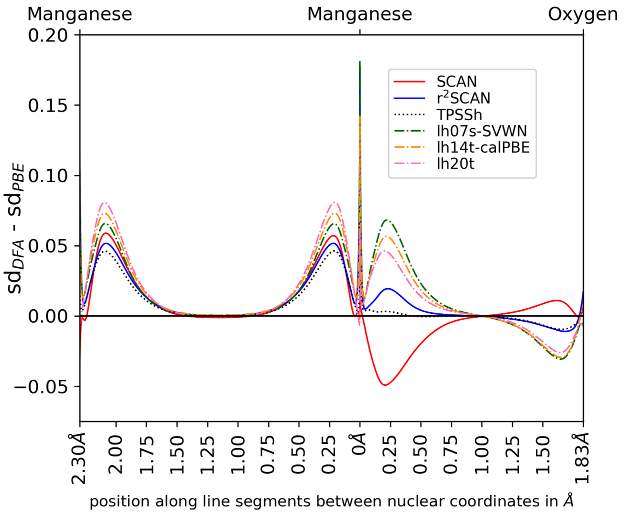

Figure S5. The spin density from PBE was subtracted from the functionals of the study and plotted along inter-nuclear line segments with distance given in Å. The high spin state of 5-VADDAF is shown.

IV. ADDITIONAL COMPUTATIONAL SETTINGS

TURBOMOLE (v. 7.4) was used for the calculations of all functionals except for lh20t and r^2SCAN for which TURBOMOLE (v. 7.6) was used. Convergence to the correct high spin and broken-symmetry states was facilitated by an initial enlargement of the HOMO-LUMO gap using a setting of '*automatic*! 0.4' and \$*scfdamp* automation settings of '$start = 1.500$, $step = 0.050$, $min = 0.400$.' The values for damping were obtained after exploring the space of settings to see at what initial damping the onset of occupation errors and failed convergence, and at which final damping does the energy begin to significantly deviate. With little guidance for settings in the available literature, it was found that suggested values for *scfdamp* from Turbomole forums and online sources typically suggested more extreme damping for transition metal complexes that is necessary for prevention of errors or incomplete convergence. The settings used above worked reliably for all complexes and functionals within the study. To ensure complete relaxation into the target state, additional calculations with less extreme settings or non-adjusted HOMO-LUMO gaps were used to analyze and inspect results. Other information or advice on achieving convergence for transition metal complexes can be given upon request. Natural population analysis (NPA) was used to analyze the convergence of calculations and to gain insight into relative differences in the ability of different methods to localize charge to metal centers. NPA results are provided above.

V. MAGNETIC HAMILTONIANS AND DATA AS GIVEN IN EXPERIMENTAL REFERENCES

Table S3. For clarity, information from the experimental references (exp. ref.) for determination of $J^{exp.}$ is provided. This includes the form of the magnetic Hamiltonian as stated explicitly or embodied within an equation for magnetic susceptibility, the values for the magnetic coupling parameter J, and the formal spin values for each ion S_1, S_2 (for some complexes, these are deduced from the provided oxidation states). For each complex, the assigned number, moniker, and citation number for the experimental article are the same as in the primary article. We used the magnetic Hamiltonian $H = -2JS_1S_2$, hence any experimental study where the 2 was absent from the Hamiltonian will have a value for J that is twice as large as the $J^{exp.}$ in the present work.

number	moniker	Hamiltonian of exp. ref.	J as printed in exp. ref.	J as in the present work	S_1	S_2	citation number
1	TIPFAZ	$H = -2JS_1S_2$	$+10(1)\ cm^{-1}$	$+10\ cm^{-1}$	3/2	3/2	[26]
2	GEFKAD	$H = -JS_1S_2$	$-6.8\ cm^{-1}$	$-3.4\ cm^{-1}$	2	2	[27]
3	KUVPEW	$H = -2JS_1S_2$	$-90\ cm^{-1}$	$-90\ cm^{-1}$	2	3/2	[28]
4	ZEQGOR	$H = -2JS_1S_2$	$-147\ cm^{-1}$	$-147\ cm^{-1}$	3/2	3/2	[29]
5	VADDAF	$H = -JS_1S_2$	$-780\ cm^{-1}$	$-390\ cm^{-1}$	3/2	3/2	[30]
6	CUAQACO2	$H = -JS_1S_2$	$-286\ cm^{-1}$	$-143\ cm^{-1}$	1/2	1/2	[31]
7	VV	$H = -2JS_1S_2$	$-107\ cm^{-1}$	$-107\ cm^{-1}$	1/2	1/2	[32]
8	YAFZOU	$H = +JS_1S_2$	$-111\ cm^{-1}$	$+55.5\ cm^{-1}$	1/2	1/2	[33]
